# Polycyclic Aromatic Hydrocarbons (PAHs) in Fired Clay Bricks Incorporating Cigarette Butts

**DOI:** 10.3390/ma14082032

**Published:** 2021-04-18

**Authors:** Halenur Kurmus, Abbas Mohajerani, Stephen Grist

**Affiliations:** 1 School of Engineering, RMIT University, Melbourne, VIC 3000, Australia; abbas.mohajerani@rmit.edu.au; 2 School of Applied Sciences, RMIT University, Melbourne, VIC 3000, Australia; stephen.grist@rmit.edu.au

**Keywords:** polycyclic aromatic hydrocarbons (PAHs), cigarette butts, recycling, fired clay bricks, environmental sustainability

## Abstract

Cigarette butts (CBs) are the most common littered waste in the world and may contain polycyclic aromatic hydrocarbons (PAHs) from the incomplete combustion of tobacco during burning. Therefore, to investigate the potential PAH residual remaining in fired clay bricks (FCBs) incorporating CBs and examine the environmental impact of utilizing toxic waste in the production of FCBs, a comprehensive PAH extraction analysis was conducted. The Soxhlet extraction method was utilized to conduct a qualitative and quantitative analysis of sixteen toxic Environmental Protection Authority (EPA) Polycyclic Aromatic Hydrocarbons (PAHs) in FCB samples incorporating CBs using gas chromatography–mass spectrometry (GC–MS). The concentrations of the mean total (Σ)PAHs for FCBs incorporating 0%, 0.5%, 1%, 1.5%, and 2% CBs by weight (wt) were found to be 0.183, 0.180, 0.242, 0.234, and 0.463 µg/mL. As expected, PAHs with higher water solubility and volatility, naphthalene, fluorene, anthracene, pyrene, fluoranthene, and chrysene were found at higher concentrations compared to lipophilic PAHs. The ΣPAH concentrations for all five FCB–CB mixes were well below the EPA Victoria solid waste hazard categorization threshold for industrial waste. Moreover, the samples were studied for their carbon content using the carbon, hydrogen, nitrogen, and sulfur (CHNS) analyzer and thermogravimetric analysis (TGA). The results confirm an almost 100% combustion process of CBs during the firing process. A content less than 0.3% suggests that all carbon within the FCB–CB mixture relatively disappeared during the firing process up to 1050 °C. However, further research regarding the emission of volatile organic compounds (VOCs) during the production of FCBs incorporating CBs should be conducted.

## 1. Introduction

Globally, over 5.4 trillion cigarettes are produced, and approximately one-third of cigarette butts (CBs) are littered into the nearby environment [1,2,3]. In Australia, CBs are the most common littered waste under the miscellaneous category, representing 91.5% [3,4]. When littered, CBs persist in the environment for up to 10 years due to the composition of the filter. The filter of a CB is made from cellulose acetate, and, although cellulose is biodegradable, the acetate content prevents the cellulose from biological decomposition in an open environment [3,5]. A study conducted in a controlled laboratory condition found that it took up to 720 days for a CB to lose 30% to 35% of its total weight while decomposing [6]. 

In cigarette smoke, it is possible to classify more than 4000 chemical components generated during burning or distilled from the tobacco [3]. Essentially, the role of the filter of the CB is to trap and absorb the particulate smoke components, consisting of more than 3500 non-volatile and semi-volatile chemical compounds. The main toxic compounds include polycyclic aromatic hydrocarbons (PAHs), nicotine, metals, catechols, carbonyls, alcohols, nicotine alkaloids, and compounds specific to Solanaceae [7,8]. Therefore, when CBs are improperly disposed of, they pose a toxicant risk to the global environment and urban and aquatic life, whereby harmful chemicals are leached [9,10]. The leachate of the toxic compounds may eventually contaminate surface water and groundwater [11,12], and ultimately bioaccumulate in the human food chain and wildlife [13,14].

Based on several years of research, Mohajerani et al. [15] proposed that the CB conundrum can potentially be eliminated by incorporating 1% CBs into 2.5% of the world’s total FCB production. Various mechanical and physical tests were conducted to determine the practicality and competency of incorporating toxic waste in the production of FCBs, and the results were promising [16,17,18,19,20]. The tests included compressive strength, density, water absorption, initial rate of absorption, efflorescence, shrinkage, energy savings, thermal conductivity, and gas emissions. The research continued with the implementation phase of recycling CBs in FCBs, whereby several sterilization methods and odor elimination techniques were reviewed, CB collection systems were explored, and an implementation procedure was proposed on an industrial scale [21]. However, an environmental issue that may evolve through the addition of CBs in the construction of FCBs is the potential leachate of heavy metals and toxic chemicals during the use and disposal of the material. In 2020, Kurmus and Mohajerani [22] conducted a comprehensive leachate study of heavy metals from FCBs incorporating various percentages of CBs. The modified FCBs were found to be below the regulatory threshold limits and categorized as non-hazardous waste when compared to the EPA Victoria industrial waste guidelines. Regarding PAHs in FCBs modified with CBs, no studies have been conducted.

PAHs in CBs are dangerous compounds produced through the incomplete combustion and pyrolysis of tobacco. They consist of two or more aromatic rings and are hydrophobic in nature with very low water solubility. PAHs are a concern, considering that they bioaccumulate and linger in the environment, with carcinogenic, teratogenic, and mutagenic properties [23,24]. In 2008, 28 PAHs were identified as priority pollutants by the National Waste Minimization Program, a project which was funded by the US Environmental Protection Agency [25]. In a study conducted by Dobaradaran et al. [12], it was found that PAHs, particularly fluorine, acenaphthylene, naphthalene, and acenaphthene, are released into the environment from freshly littered CBs. In addition to CBs, PAH contamination in soil is a common issue, because soil is less receptive to chemical and biological degradation and the PAHs tend to absorb tightly into the organic matter present in the soil. Moreover, the recalcitrance of PAHs towards treatment increases with prolonged aging time, promoting the sequestration of PAH molecules into micropores [26].

Thus, the aim of this study was to conduct a comprehensive evaluation of PAHs in FCBs incorporated with CBs to examine the potential environmental impact of utilizing this toxic waste in the production of FCBs. In this study, a direct Soxhlet extraction method was employed using dichloromethane as the solvent. The aim of the extraction process is to effectively remove the analyte (PAHs) from its matrix, with minimal solvent usage [27]. The extract was analyzed for sixteen US EPA priority PAH compounds using gas chromatography–mass spectrometry (GC–MS) in accordance with the US EPA Method 3540C [28]. The PAH concentrations were then compared to the Environmental Protection Authority (EPA) Victorian solid industrial waste hazard categorization and management thresholds [29] to assess the suitability of FCBs incorporating CBs. Furthermore, the FCB samples incorporating CBs were studied for their carbon content using the carbon, hydrogen, nitrogen, and sulfur (CHNS) analyzer and thermogravimetric analysis (TGA).

## 2. Materials and Methods

### 2.1. Materials and Brick Manufacturing Methodology

Soil and CBs are the raw materials that were used in this study. A sieve analysis was conducted to classify the soil, and, according to unified soil classification, the soil was categorized as sandy silty clay (MC) [30]. The soil material was obtained from PGH Bricks and Pavers (Melbourne, Australia) and the CBs, of distinct brands and sizes, were provided by Butt Out Australia Pty Ltd (Melbourne, Australia). The raw materials, CBs and soil, were initially oven-dried at 105 °C for 24 h. Various percentages (0%, 0.5%, 1%, 1.5%, and 2% CBs by weight (wt)) of CBs were then added and uniformly mixed with the soil for 25 min in a mechanical mixer at a moisture content of 15.5%. The fast-mixing process caused the CBs to shred and homogenously integrate into the soil mixture. Prior to being placed in the oven at 105 °C for 24 h, the samples were compacted in cylindrical molds at a compaction pressure of 240 kPa. The final step involved firing the brick samples in the furnace at 1050 °C for 3 h. For each batch, three replicate samples were prepared, tested, and the mean value was reported. The compressive strength, water absorption, initial rate of absorption, shrinkage, and density property results for the FCBs incorporating 0%, 0.5%, 1%, 1.5%, and 2% CBs by wt. can be found in Kurmus and Mohajerani [19].

The chemical and mineralogical composition of the CB brick samples were determined through AXS D8 Endeavor wide-angle X-ray diffractometer (XRD) (Bruker, MA, USA) and AXS S4 Pioneer spectrometer X-ray fluorescence (XRF) (Bruker, MA, USA). Powder samples were prepared by crushing and sieving the FCB samples incorporating CBs to 10 µm in size. The XRD was operated at 45 kV and 35 mA in reflection scanning mode from 6° to 90° for a total scan time of 29 min.

### 2.2. Carbon Analysis

**Figure 1:**
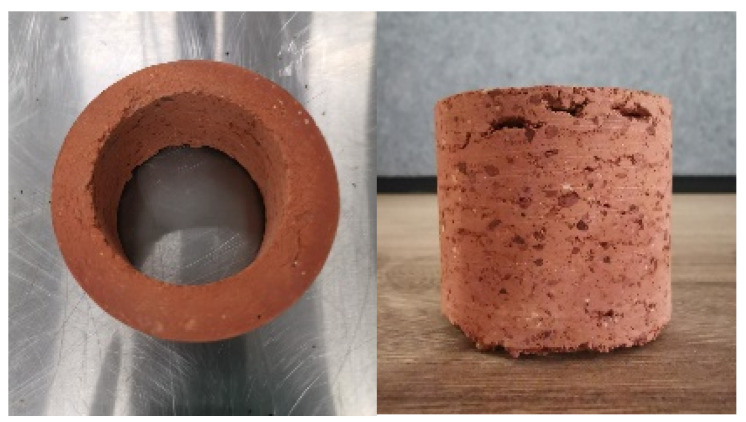
The core of the brick extracted for CHNS analysis.

The CHNS-2400 Elemental Analyser (PerkinElmer, MA, USA) was used to analyze the total carbon, hydrogen, nitrogen, and sulfur content in FCBs incorporating 0%, 0.5%, 1%, 1.5%, 2%, and 5% CBs by wt. The purpose of this investigation was to determine whether carbon was trapped within the core of the brick structure from the potential incomplete combustion of CBs during the firing process. Therefore, for this experiment, only the extracted core samples were analyzed. The core of the 100-mm-diameter bricks was extracted using a 50-mm drill-saw, as shown in Figure 1. The extracted core bricks were crushed and sieved through a 1.14-mm sieve, and the quadrat sampling method was employed to select a homogeneously distributed sample for testing.

CHNS analysis was conducted by combusting samples of 5 mg at a high temperature of 850 °C in a chamber in the presence of oxygen. The occurrence of oxygen allows individual elements to combine with oxygen and form gaseous byproducts and water vapor such as nitrogen (N_2_), sulfur dioxide (SO_2_), water (H_2_O), and carbon dioxide (CO_2_). The gases are then captured in the gas control zone and homogenized. The homogenized gases are depressurized through a column, and the separation approach is used to elute the gases, which are measured and detected by a thermal conductivity detector. In this study, three replicate samples were prepared, and the mean average was reported.

Thermogravimetric analysis (TGA) and differential thermal analysis (DTA) were performed using the TGA 8000 Autosampler (PerkinElmer, MA, USA) to monitor the mass loss of the brick samples incorporating CBs. For the thermal analysis, the samples were heated from 30 °C to 850 °C at a constant heating rate of 20 °C/min in a 15 mL-per-min flow of nitrogen. For the purpose of this investigation, the brick samples were crushed to a particle size of <75 µm. All samples were tested under the same conditions.

### 2.3. Polycyclic Aromatic Hydrocarbon (PAH) Analysis

#### 2.3.1. Extraction Method

The Soxhlet extraction technique is a widely employed method to extract PAHs from sediments and soils [31]. Therefore, the brick samples were extracted in accordance with the USEPA Soxhlet Extraction Method 3540C [28]. In the basic Soxhlet extraction test, 15 g of crushed brick samples of particle size <1 mm is placed into a cellulose thimble, which is then extracted using 300 mL of solvent via the reflux cycle. In this case, dichloromethane was used as the solvent. The whole assembly was heated for 8 h using an isomantle. The extracts from the Soxhlet extractor were concentrated to 10 mL using the rotary evaporator and then diluted. The concentrated samples were mixed with five isotopically labeled internal standard solutions to make a final concentration of 10 µg/mL. The samples were stored at 4 °C for GC–MS measurement.

#### 2.3.2. Standards

PAH calibration standards, including five types of isotopically labeled internal standards and 16 types of PAHs, were used for quantification. The PAH mix (2000 µg/mL) was purchased from Supelco and included acenaphthene, dibenz[*a*,*h*]anthracene, anthracene, chrysene, benz[*a*]anthracene, benzo[*b*]fluoranthene, fluorene, benzo[*k*]fluoranthene, naphthalene, benzo[*a*]pyrene, phenanthrene, benzo[*ghi*]perylene, pyrene, fluoranthene, acenaphthylene, and indeno [1,2,3-*cd*]pyrene. The internal standards (2000 µg/mL) were also purchased from Supelco and included benz[*a*]anthracene-d12 solution, chrysene-d12 solution, naphthalene-d8 solution, perylene-d12 solution, and phenanthrene-d10 solution.

Five-point standard calibration solutions were prepared to ensure the accuracy and precision of the PAHs for quantitative and qualitative analysis. The standard calibration solutions were measured at levels ranging from 1.25 to 25 µg/mL and internal standards of 10 µg/mL. Each analytical series included one blank test. The 16 PAHs and five internal standards in the samples were defined by the precursor ions and retention time, as shown in Table 1.

#### 2.3.3. GC–MS Analysis

A GC–MS system connecting a gas chromatograph with a triple-quadrupole mass spectrometer and equipped with an auto-sampler and sample preparation robot (Agilent 7000 Series GC–MS triple quad system) was used to analyze the PAH compounds. Two Agilent DB-5MS columns 5 m long and 0.25 mm in diameter were used with mid-column backflush. The multi-mode injector was operated in solvent elimination mode with injection at 5 °C, ramping at 600 °C/min to 325 °C. The oven temperatures were between 5 and 325 °C after an initial holding time of 1 min at 25 °C/min. To attain an initial oven temperature of 5 °C, CO_2_ was used as the carrier gas for cooling at a constant flow of 3 mL/min. The cryogenics mode offered the ultimate conditions for the solvent vent mode. The injection volume was set to 0.2 μL and the electronic energy to 70 eV. Each run took approximately 17 min.

## 3. Results and Discussion

### 3.1. Characterization of Brick Samples

**Figure 2:**
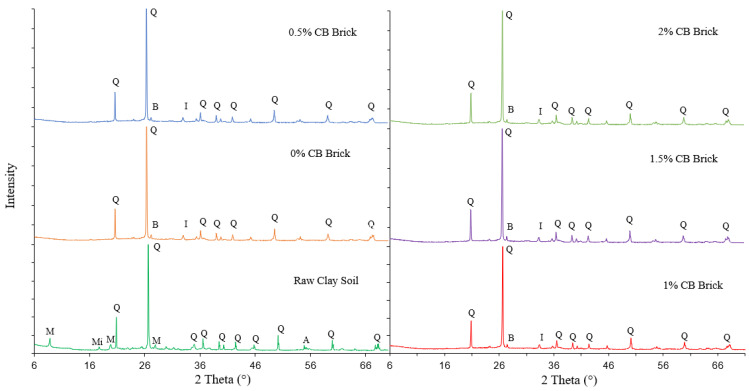
XRD spectra of raw clay soil and FCBs incorporating 0%, 0.5%, 1%, 1.5%, and 2% CBs. Q: Quartz, M: Muscovite, Mi: Mikasite, A: Albite, B: Bismuth oxide, I: Iron chromium oxide.

The chemical composition of the raw clay material and bricks incorporating CBs is presented in Table 2. The data provided in Table 2 show that the major compounds in the raw clay are silicon dioxide (SiO_2_), saluminum oxide (Al_2_O_3_), iron(III) oxide (Fe_2_O_3_), and potassium oxide (K_2_O). Similarly, the chemical compounds found in the FCB samples incorporating various percentages of CBs were SiO_2_, Al_2_O_3_, Fe_2_O_3_, and K_2_O. The XRD analysis results for the raw clay soil and FCBs specimens incorporating CBs are presented in Figure 2. The XRD peak at 26.7° indicates that the soil and brick samples incorporating CBs are mainly made of quartz, which is consistent with the XRF data presented in Table 2, demonstrating the presence of a large amount of silica. The second-highest peak corresponding to the raw clay soil is muscovite, a hydrated phyllosilicate mineral of aluminum and potassium, which are present in quantities of 19.95% and 2.87%, respectively, in the XRF results.

### 3.2. Carbon Analysis

CBs contain a high content of carbon, which may stem from the unburnt tobacco, the wrapping paper, or the cellulose acetate filter, and when they are incorporated into the production of FCBs, carbon may be trapped within the brick structure. Therefore, to investigate whether the CBs within the brick structure completely burn off during firing, a CHNS and TG analysis was conducted. The results pertaining to organic CHNS contents and the mass loss from the TGA are shown in Table 3. As expected, FCBs incorporating 5% CBs contain the preeminent carbon content of 0.29% due to the high CB content in the FCB, and, although it is the highest, the effect will not be significant because of the very low percentage. A carbon content less than 0.3% suggests that all carbon within the FCB–CB mixture relatively disappeared during the firing process up to 1050 °C. As displayed in Table 3, the samples contained a high content of nitrogen, which varied from 3.74% to 6.36% in the FCB samples containing between 0% and 5% CBs. This can be explained by the fact that nitrogen is the most abundant element in the atmosphere, and it is present in organic materials—in this case, soil—which then become available to plants, such as tobacco [32]. For the hydrogen and sulfur contents, all samples were found to contain 0%.

The TGA results of the brick samples incorporating 0%, 0.5%, 1%, 1.5%, and 2% CBs demonstrated mass losses of 0.41%, 0.47%, 0.54%, 0.65%, and 0.68%. The weight loss between 50 and 120 °C is mainly due to the removal of moisture, and the mass loss from 230 to 430 °C is primarily due to the decomposition and combustion of organic matter remaining from the incomplete combustion of the CBs or soil during the firing process at 1050 °C [33]. While the CHNS analyzer solely measures the carbon content, the mass loss from the TGA comprises both the carbon (combustion of organic matter) and moisture content, which is the source of the deviation between the results.

### 3.3. Polycyclic Aromatic Hydrocarbon (PAH) Analysis

**Figure 3:**
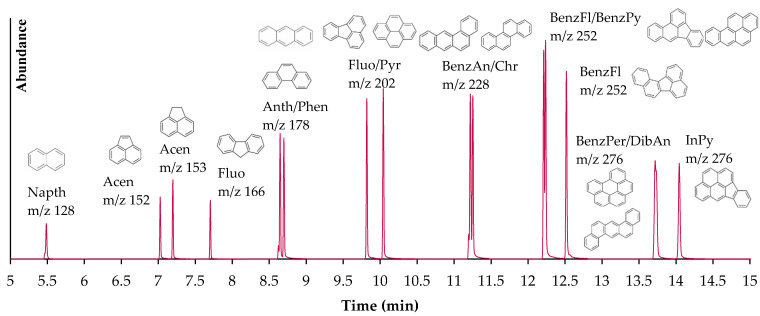
GC–MS chromatogram at 25 µg/mL in DCM for 16 PAHs.

The selected GC–MS quantification ion chromatograms of 16 PAHs in a standard mixture can be seen in Figure 3. The chromatogram displays excellent sensitivity, separation, and peak shape. The internal standards naphthalene-d8, phenanthrene-d10, perylene-d12, benz[*a*]anthracene-d12, and chrysene-d12 were used to quantify naphthalene, acenaphthene, acenaphthylene, anthracene, fluoranthene, fluorene, phenanthrene, pyrene, benz[*a*]anthracene, chrysene, benzo[*b*]fluoranthene, benzo[*k*]fluoranthene, benzo[*ghi*]perylene, benzo[*a*]pyrene, dibenz[*a*,*h*]anthracene, and indeno[1,2,3-*cd*]pyrene. The results behaved linearly over a concentration range from 1.25 µg/mL to 25 µg/mL for all 16 PAHs.

**Figure 4:**
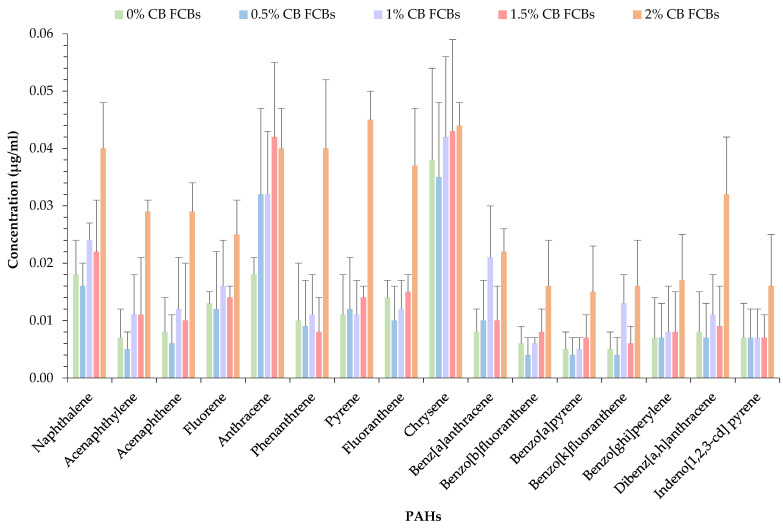
Results of PAHs found in FCBs incorporating 0%, 0.5%, 1%, 1.5%, and 2% CBs.

The results of the FCBs incorporating CBs for 16 PAHs are illustrated in Figure 4. Currently, there are no similar studies involving the extraction of PAHs from FCBs or wastes recycled in FCBs to compare the results. However, research on the emission of PAHs from littered CB samples has been performed, whereby Dobaradaran et al. [12] found a mean ΣPAH concentration level of 24.6 µg/mL in freshly smoked CB samples and 20.4 µg/mL in city CB samples. When compared to the current study, the mean ΣPAH content in FCBs incorporating 0%, 0.5%, 1%, 1.5%, and 2% CBs was found to be at concentrations of 0.183, 0.180, 0.242, 0.234, and 0.463 µg/mL. It is apparent by incorporating CBs into the production of FCBs that a major decrease in PAH emissions into the environment is achievable. However, the major decrease in PAH concentrations may be due to several reasons, including the processes involved in manufacturing the FCB–CB samples. The brick manufacturing methods include oven drying, mixing (with water and soil), compacting, and firing, which may all result in the dissolution or evaporation of PAHs. This is further confirmed by the revised study [12], which found naphthalene to have the highest concentration level in both collected CBs from city streets and freshly smoked CB samples. In this current study, naphthalene was the second/third most prominent PAH for all sample types. The variations may be from the higher solubility of naphthalene, and thus the potential dissolution or evaporation of the PAH during the production processes, those factors being high temperatures or high-speed mixing.

Another study that involved the leachate of ΣPAHs from CBs into various water types found concentration levels ranging from 3.0 to 5.0 µg/L, 3.3 to 5.5 µg/L, and 3.9 to 5.7 µg/L for river, tap, and deionized waters, respectively [23]. The concentrations of this study are fairly low, as the study involved determining the leachate of PAHs from CBs in water samples through liquid–liquid extraction, compared to our study, which involved solid-phase extraction (SPE). The SPE method has a greater detection of sensitivity and is effective in extracting highly soluble substances. In addition to the method, the type of solvent used for extraction is critical. The initial solvent used for the Soxhlet extraction procedure was hexane–acetone (50:50). This decision was based on the theory of like dissolves like. Various studies found that dichloromethane (polar) resulted in low recoveries for all PAH (nonpolar) compounds when used as an extraction solvent, whereas hexane–acetone (nonpolar–polar) was primarily effective [34,35]. However, in our study, hexane–acetone resulted in chromatograms with poor sensitivity, separation, and peak shape. For this reason, the solvent was changed to dichloromethane. Dichloromethane resulted in excellent chromatograms and high PAH recoveries, as shown in Figure 3.

The sample with the highest ΣPAH concentration was in the FCB samples incorporating 2% CBs. The order of the ΣPAH concentrations in the FCBs according to the percentage of CBs incorporated was as follows: 2% CBs > 1% CBs > 1.5% CBs > 0% CBs > 0.5% CBs. The sample with the highest CB content exhibited the highest ΣPAH content as expected, the concentration of the 16 PAHs ranging from 0.015 to 0.044 µg/mL. However, it must be pointed out that the main raw material employed in preparing the brick samples was soil, and soil contamination is a common phenomenon. PAHs are known to absorb tightly to the organic matter in soil, rendering them less susceptible to biological and chemical degradation. For example, Saim et al. [27] extracted 1623 µg/mL of ΣPAHs from coal-derived contaminated land soil using the Soxhlet extraction method, while Yu et al. [36] extracted 582 µg/kg (0.582 µg/mL) from soils in the Guiyu area of China. Therefore, it is necessary to mention that the extracted PAH concentrations in the brick samples may be a result of the soil being contaminated, in addition to the CBs.

**Figure 5:**
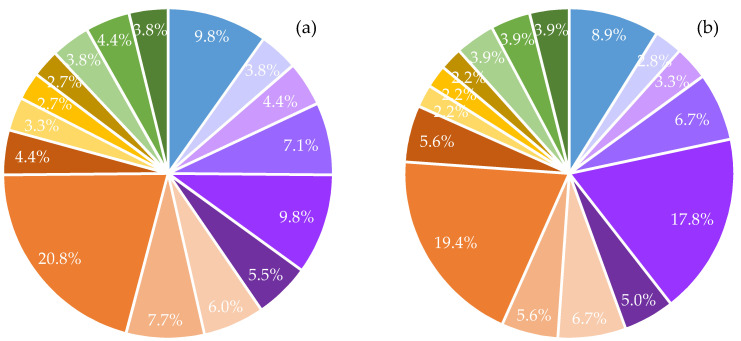

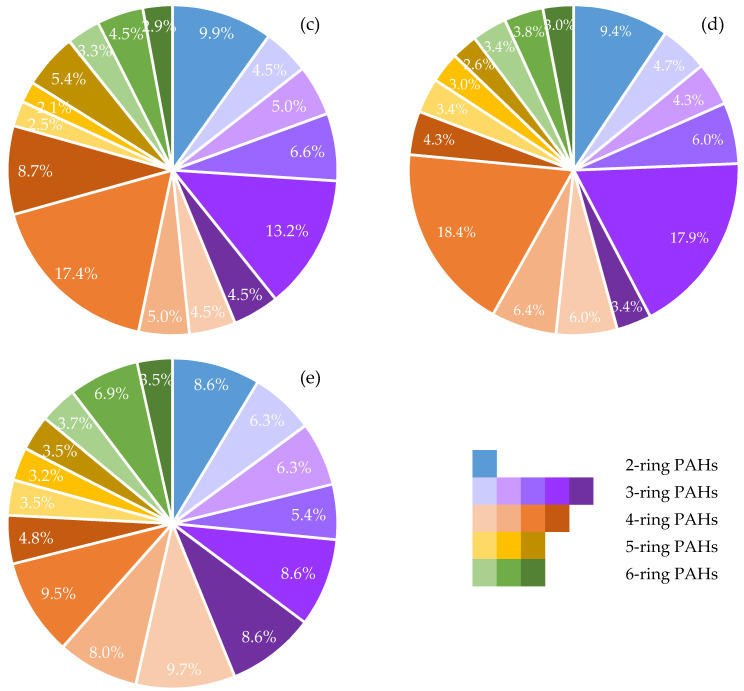
Distribution of 2-3-4-5-6-ring PAHs (% of sum of 16 PAHs) in FCBs incorporating (**a**) 0%, (**b**) 0.5%, (**c**) 1%, (**d**) 1.5%, and (**e**) 2% CBs.

As shown in Figure 5, the FCB–CB samples contained a high concentration of PAHs predominately with 2-3-4-rings compared to those with 5-6-rings. Chrysene and anthracene constituted 9.5% to 20.8% and 8.6% to 17.9%, respectively, of the mean ΣPAHs extracted from the brick samples. As expected, naphthalene, fluorene, anthracene, pyrene, fluoranthene, and chrysene, which are PAHs with higher water solubility and volatility, were found at higher concentrations compared to lipophilic PAHs, which include benzo[*b*]fluoranthene, benzo[*k*]fluoranthene, benzo[*ghi*]perylene, benzo[*a*]pyrene, dibenz[*a*,*h*]anthracene, and indeno[1,2,3-*cd*]pyrene. The lowest concentration was found at 0.004 µg/mL for PAHs with 5-rings. In general, the ΣPAH concentrations were the lowest in PAHs with 5-rings, followed by 6-rings, 2-rings, 3-rings, and 4-rings for all sample types.

According to the Industrial Waste Resource Guidelines [29], if the total PAH concentration is below 50 µg/mL, the waste is considered industrial waste (IW). IWs are not considered prescribed wastes; therefore, they do not require control or management when disposed of to landfills and will be accepted at solid inert landfills (non-putrescible) or municipal solid waste landfills (putrescible). In this case, the mean ΣPAHs for FCBs incorporating 0%, 0.5%, 1%, 1.5%, and 2% CBs range between 0.180 and 0.463 µg/mL and are significantly below the limit of 50 µg/mL.

## 4. Conclusions

In the present study, the concentration levels of 16 EPA-PAHs extracted from fired clay brick (FCB) samples, incorporating various percentages of cigarette butts (CBs), were evaluated. The results show that lower-molecular-weight PAHs (light PAHs), including naphthalene, fluorene, anthracene, pyrene, fluoranthene, and chrysene, with higher volatility and lower lipophilicity, were released at very low concentrations compared to the higher-molecular-weight PAHs (heavy PAHs) from FCB–CB samples.

Among the detected PAHs, chrysene and anthracene dominated the emissions and constituted 9.5% to 20.8% and 8.6% to 17.9%, respectively, of the mean ΣPAHs extracted from the brick samples. Lipophilic PAHs including benzo[*b*]fluoranthene, benzo[*k*]fluoranthene, benzo[*ghi*]perylene, benzo[*a*]pyrene, dibenz[*a*,*h*]anthracene, and indeno[1,2,3-*cd*]pyrene demonstrated lower PAH concentrations due to their higher weights. The mean ΣPAH content in FCBs incorporating 0%, 0.5%, 1%, 1.5%, and 2% CBs was found in concentrations of 0.183, 0.180, 0.242, 0.234, and 0.463 µg/mL. As expected, the highest ΣPAH concentration was found in FCB samples incorporating 2% CBs. The concentrations of the mean ΣPAH for all samples were well below the EPA solid waste hazard categorization threshold limit for industrial waste.

Carbon, hydrogen, nitrogen, and sulfur (CHNS) and thermogravimetric (TG) analysis were conducted on the FCB–CB samples to investigate whether the CBs are completely combusted during the firing process. The results confirm an almost 100% combustion process during the firing process. A content less than 0.3% suggests that all carbon within the FCB–CB mixture relatively disappeared during the firing process up to 1050 °C.

It can be concluded that the results presented in this study clarify the issues raised regarding the possible release of toxic PAHs into the environment during the use and disposal of FCBs incorporating CBs or the potential storage of carbon in the FCB–CB mixture. However, further research regarding the emission of volatile organic compounds (VOCs) during the storage of CBs and during the mixing, oven-drying, and firing process of producing FCBs incorporating CBs should be conducted. In addition, potential deodorization and sterilization methods of CBs should be examined and implemented.

## Figures and Tables

**Table 1:** Physico-chemical characteristics and optimized MRM acquisition parameters of target PAHs.

Compound	Retention Time (min)	Precursor Ion (m/z)	Product Ion (m/z)	No. of Aromatic Rings	Water Solubility (mg/L)
Naphthalene	5.51	128	128/128	2	31.69
Naphthalene-d8 (IS)	5.49	136	108/136		-
Acenaphthylene	7.07	152	152/152	3	3.93
Acenaphthene	7.24	153	153/153	3	3.93
Fluorene	7.73	166	165/166	3	1.68–1.98
Anthracene	8.66	178	178/178	3	0.0446
Phenanthrene	8.71	178	178/178	3	1–1.6
Phenanthrene-d10 (IS)	8.64	188	160/188		-
Pyrene	9.83	202	202/202	4	0.129–0.165
Fluoranthene	10.05	202	202/202	4	0.206
Chrysene	11.22	228	228/228	4	0.0015–0.0022
Chrysene-d12 (IS)	11.20	240	236/240		-
Benz[*a*]anthracene	11.26	228	228/228	4	0.011
Benz[*a*]anthracene-d12 (IS)	11.23	240	236/240		-
Perylene-d12 (IS)	11.75	241	241/242		-
Benzo[*b*]fluoranthene	12.21	252	252/252	5	0.0012
Benzo[*a*]pyrene	12.24	252	252/252	5	0.0038
Benzo[*k*]fluoranthene	12.52	252	252/252	5	0.0008
Benzo[*ghi*]perylene	13.72	276	276/276	6	0.00026
Dibenz[*a,h*]anthracene	13.74	278	278/278	6	0.0005
Indeno[1,2,3-*cd*] pyrene	14.05	276	276/276	6	0.062

**Table 2:** Chemical composition of raw clay soil and brick samples incorporating CBs.

Component	Raw Clay Soil (%)	Percentages of CBs by wt.				
		0%	0.50%	1%	1.50%	2%
Na_2_O	2.46	0.5	0.4	0.4	0.4	0.5
MgO	1.62	1.1	1.1	1.2	1.1	1.2
Al_2_O_3_	19.95	16.8	16	16.5	16.3	16.8
SiO_2_	62.84	53.8	52.6	54.8	53.2	55.3
P_2_O_5_	-	0.2	0.2	0.2	0.3	0.3
K_2_O	4.87	3.5	3.4	3.4	3.5	3.5
CaO	0.27	0.2	0.2	0.3	0.3	0.4
TiO_2_	1.19	0.9	0.9	0.9	0.9	0.9
Fe_2_O_3_	6.15	6.9	6.9	6.8	6.8	6.7

**Table 3:** Concentration of carbon, hydrogen, nitrogen, and sulfur content and mass loss in bricks incorporating 0%, 0.5%, 1%, 1.5%, 2%, and 5% CBs by wt.

Mixture Identification (%)	CHNS Results				TGA Results
	Carbon (%)	Hydrogen (%)	Nitrogen (%)	Sulfur (%)	Mass Loss (%)
CB (0)	0.02	Not detected	3.74	Not detected	0.41
CB (0.5)	0.03	Not detected	4.74	Not detected	0.47
CB (1.0)	0.02	Not detected	6.01	Not detected	0.54
CB (1.5)	0.01	Not detected	6.16	Not detected	0.65
CB (2.0)	0.04	Not detected	4.88	Not detected	0.68
CB (5.0)	0.29	Not detected	6.36	Not detected	-

## Data Availability

Data sharing is not applicable to this article.
